# Self Reported Childhood Difficulties, Adult Multimorbidity and Allostatic Load. A Cross-Sectional Analysis of the Norwegian HUNT Study

**DOI:** 10.1371/journal.pone.0130591

**Published:** 2015-06-18

**Authors:** Margret Olafia Tomasdottir, Johann Agust Sigurdsson, Halfdan Petursson, Anna Luise Kirkengen, Steinar Krokstad, Bruce McEwen, Irene Hetlevik, Linn Getz

**Affiliations:** 1 Department of Family Medicine, University of Iceland and Primary Health Care of the Capital Area, Reykjavik, Iceland; 2 General Practice Research Unit, Department of Public Health and General Practice, Norwegian University of Science and Technology (NTNU), Trondheim, Norway; 3 Department of General Practice, UiT The Arctic University, Tromsø, Norway; 4 HUNT Research Centre, Department of Public Health and General Practice, Norwegian University of Science and Technology (NTNU), Levanger, Norway; 5 Laboratory of Neuroendocrinology, The Rockefeller University, New York, New York, United States of America; Central South University, CHINA

## Abstract

**Background:**

Multimorbidity receives increasing scientific attention. So does the detrimental health impact of adverse childhood experiences (ACE). Aetiological pathways from ACE to complex disease burdens are under investigation. In this context, the concept of *allostatic overload* is relevant, denoting the link between chronic detrimental stress, widespread biological perturbations and disease development. This study aimed to explore associations between self-reported childhood quality, biological perturbations and multimorbidity in adulthood.

**Materials and Methods:**

We included 37 612 participants, 30–69 years, from the Nord-Trøndelag Health Study, HUNT3 (2006–8). Twenty one chronic diseases, twelve biological parameters associated with allostatic load and four behavioural factors were analysed. Participants were categorised according to the self-reported quality of their childhood, as reflected in one question, alternatives ranging from ‘very good’ to ‘very difficult’. The association between childhood quality, behavioural patterns, allostatic load and multimorbidity was compared between groups.

**Results:**

Overall, 85.4% of participants reported a ‘good’ or ‘very good’ childhood; 10.6% average, 3.3% ‘difficult’ and 0.8% ‘very difficult’. Childhood difficulties were reported more often among women, smokers, individuals with sleep problems, less physical activity and lower education. In total, 44.8% of participants with a very good childhood had multimorbidity compared to 77.1% of those with a very difficult childhood (Odds ratio: 5.08; 95% CI: 3.63–7.11). Prevalences of individual diseases also differed significantly according to childhood quality; all but two (cancer and hypertension) showed a significantly higher prevalence (p<0.05) as childhood was categorised as more difficult. Eight of the 12 allostatic parameters differed significantly between childhood groups.

**Conclusions:**

We found a general, graded association between self-reported childhood difficulties on the one hand and multimorbidity, individual disease burden and biological perturbations on the other. The finding is in accordance with previous research which conceptualises allostatic overload as an important route by which childhood adversities become biologically embodied.

## Introduction

Most consultations with adults in primary care involve more than one health problem or disease [1,2]. Multimorbidity, defined by WHO as being affected with two or more chronic health conditions [3], has received increased recognition over the past years [4,5] and has even been termed one of the major medical challenges of the 21st century [3,6]. Recent research sheds light on various aspects of multimorbidity, mostly focusing on prevalence data [5,7–10] and specific patterns of clustering [11–13]. Multimorbidity increases with age [7,8,14] and is more common in lower socioeconomic groups [8,15,16]. Beyond this, scientific knowledge pertaining to multimorbidity is still incomplete [10,17].

Multimorbid disease clusters tend to defy diagnostic categories within the ‘somatic’ and ‘mental health’ domains respectively, and typically also transgress this dichotomy [10,11]. This evokes the question whether multimorbidity ought to be seen as an artefact of the reigning biomedical classification systems, sometimes referred to as medical ‘silo’ thinking [10,18–20].

Recognizing multimorbidity as a fundamental challenge to both medical theory and practice, authoritative voices have called for a shift from fragmented, disease-oriented medical care to an integrative ‘person-focused’ or ‘person-centered’ care [21,22]. Irrespective of on-going controversies relating to the practical delivery of clinical care, the link between low socio-economic status and multimorbidity has actualized a scientific interest in potential underlying causes of ill health in general [15,17,20]. Using terms such as ‘the causes behind the causes’ and ‘the biology of disadvantage’ researchers draw scientific attention to the general impact of relational and socio-political factors which undermine human health [10,23].

The technological capacity to explore bio-molecular mechanisms which might link lifetime experiences to human health and disease has evolved rapidly during recent years. Researchers focus on various pathways or markers, such as immune mechanisms [24–27], autonomic imbalance [27–31], endocrine stress responses [32–34], epigenetic mechanisms [35,36], and telomere maintenance [37,38]. This reflects how stress exerts its effects on various biological subsystems and indicates the relevance of exploring the human physiological adaptive systems as a complex whole. The concept of allostasis (gr: stability through change) [39] is based on such an integrative perspective, as previously described [10,23]. Essentially, *allostasis* refers to a living organism’s physiological ability to guard its integrity (including cellular homeostasis) when encountering challenges and stressors. *Allostatic load* denotes the cumulative impact of strain on the organism over time, while allostatic *overload* denotes a ‘red flag’ physiological risk scenario, where the organism’s adaptive and restorative capacity is overtaxed to such an extent that adaptability and flexibility decline prematurely [39–41]. Allostatic overload results in a gradual loss of physiological flexibility, initially reflected by subtle but wide-spread physiological perturbations and an increased risk of complex disease development, informed by congenital and acquired susceptibilities [10].

The trajectory from adverse childhood experiences to health problems in adult life has received increasing scientific attention since the late 1990s. The US Adverse Childhood Experiences Study represented a milestone as it documented a linear relationship between the number of adversity categories in childhood and morbidity-burden in adult life, both in the somatic and mental domains [42,43]. Associations between adverse childhood experiences and health problems in adult life (somatic and psychiatric conditions, including addictive behaviours and sleep problems) have later been confirmed in various contexts [44–54]. These studies have typically focused on predefined adverse experiences, including different forms of abuse, neglect and dysfunctional households [50,54–59]. Increasing evidence links adverse childhood experiences to future health problems with reference to allostatic overload [60–63]. To our knowledge, the association between a *subjective*, *global* evaluation of the childhood and adult health has not been examined.

### Research hypothesis

**Fig 1:**
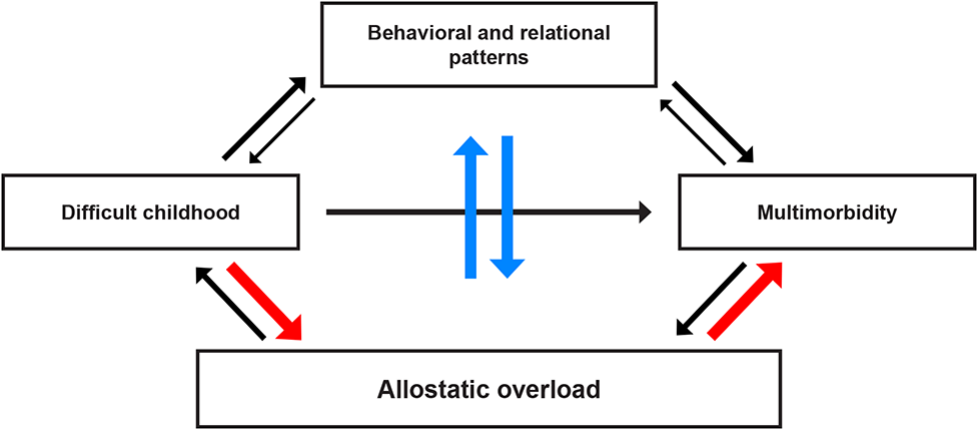
Model illustrating the hypothesized links between childhood difficulties and multimorbidity. All arrows indicate potential pathways connecting adverse childhood experiences to multimorbidity. The solidity of the arrows reflect the proposed relative impact of the illustrated factors. Our main hypothesis is indicated by the red arrows leading from a difficult childhood to multimorbidity through allostatic overload. The blue arrows indicate a presumed impact of behavioural and relational patterns in this development. The black arrows reflect additional pathways that might play a significant but generally more limited role.

In light of the documented association between adverse childhood experiences and health problems, as well as conceptual and empirical links between childhood difficulties and allostatic overload, we outline a framework for our hypothesis, based on our understanding of the topic and the research literature (Fig 1). The aim of the present study was to explore the connections indicated in the model by studying the association between experience of childhood and multimorbidity in adult life, taking into account the possible effect of behavioural factors as well as markers of allostatic overload.

## Study Population and Methods

The Nord-Trøndelag Health Study (HUNT) is a renowned, population based study whose third wave, HUNT3, was carried out in 2006–2008. Every adult living in Nord-Trøndelag County, Norway, was invited to participate and 54% accepted participation [64]. The HUNT3 population has been considered fairly representative of the Norwegian population. It is ethnically homogenous, and since Nord-Trøndelag lacks large cities, the social inequalities in the HUNT population might be smaller than for Norway in general [64,65].

The HUNT3 data were collected through questionnaires, physical examinations and blood samples. For the present analysis people aged 30–69 years who answered the question regarding childhood experience were included, in total 37 612 participants with participation rate of 58% (missing 373 individuals or 1% that did not answer regarding childhood experience) [64]. The youngest age groups were somewhat underrepresented, with only 31% participation rate for people aged 20–29 years [66]. They were therefore excluded from the present analyses along with people aged 70 years or more in whom multimorbidity is highly prevalent due to age [7].

### Assessment of childhood difficulties in HUNT3

The overall quality of the respondents’ childhood was addressed in HUNT3 by one single question with five fixed response alternatives, referring to the respondent’s subjective, global perception of his/her childhood. Our *childhood experience question* was phrased (here translated to English): ‘When you think about your childhood, would you describe it as’: ‘Very good–good–average–difficult–very difficult’. The question appeared among relatively neutral questions related to everyday topics such as intake of dairy products and living with pets in childhood (questionnaire accessible at www.hunt.no). We worded the question with respect to the local linguistic and cultural context, supported by a linguist.

### Assessment of multimorbidity, behavioural patterns and allostatic parameters

We defined multimorbidity as two or more coinciding chronic diseases or conditions in accordance with international consensus [3,18]. For a fair evaluation of multimorbidity, data on at least twelve relevant chronic diseases are needed [9]. Our analysis includes 21 chronic diseases or conditions, as has previously been described in more detail [10]. Any case of missing data was defined as absence of the disease in question.

Regarding behavioural patterns, we included daily smoking and mean number of cigarettes, sleep problems and physical activity. Daily smoking was defined as use of cigarettes, cigars, pipes and/or snuff daily. Physical activity was measured as a combination of light and hard exercise during the last year, measured in hours as no activity, less than three hours of light activity, more than three hours of light but less than one hour of hard activity and finally more than one hour of hard activity per week.

The HUNT3 database lacks direct data on socioeconomic status (SES). Information regarding educational level was however accessible for 76% of our respondents who had also completed the HUNT2 survey 10 years earlier [64]. This was used as a marker of adult SES.

Sleep problems were defined as difficulty falling asleep, waking up repeatedly during the night or waking too early and not being able to fall asleep again, several times per week for the last month.

To address the possibility of recall bias associated with depression, multimorbidity analyses were also performed after adjusting for indications of current depression, defined as eight or more points on the Hospital Anxiety and Depression Scale (HADS). Multimorbidity and experience of childhood were also compared between depressed and non-depressed groups, respectively.

Allostatic load parameters have been classified as *primary* (being mostly chemical messengers in response of short term stress), *secondary* (reflecting cumulative actions of primary parameters in a tissue/organ-specific manner) and *tertiary* (emerging as clinical diseases or disorders) [67,68]. Somewhat different parameters have been applied and combined to estimate allostatic load in different studies [69]. Our analysis includes twelve secondary allostatic parameters.

For the estimation of systolic and diastolic blood pressure, heart rate and pulse pressure, HUNT3 participants using antihypertensive medication or diagnosed with cardiovascular disease were excluded to avoid medication bias. Likewise, participants reporting diabetes were excluded from estimation of serum glucose. Similar precautions were not possible for cholesterol, as information on cholesterol-lowering medication was unavailable.

### Statistical analyses

Descriptive analyses were stratified according to childhood experience. The categorical variables were expressed as frequencies with percentages and continuous variables as means with standard deviations. Differences between childhood groups with p-trends were estimated with Mantel-Haenszel test for linear association and ANOVA test for linearity as appropriate.

Prevalences were estimated for the number of diseases in each group of childhood experience with 95% confidence intervals (CI). The same was performed for individual diseases. Mantel-Haenszel test for linear association was used to test if disease prevalence followed a gradient from very good to very difficult childhood.

Binomial logistic regression was used to assess the odds ratios (OR) of multimorbidity according to childhood experience. All logistic calculations were adjusted for age and gender. Behavioural and biological factors were then introduced to the model, both individually and in different combinations. Participants with missing data regarding allostatic parameters were excluded in all logistic regression models, but missing data on behavioural factors were coded as an additional group for precise comparison between models.

Parameters pertaining to allostatic load were analysed according to childhood experience for each gender. Means were estimated with participants reporting a very good childhood as the reference group. Deviances from the mean according to each group of childhood experience, as well as p-trend, were subsequently estimated with linear regression after adjusting for age.

SPSS statistical program (version 20) was used for all analyses.

### Ethics Statement

Each participant in the HUNT Study signed a written consent regarding the screening and the use of data for research purposes. The study was approved by the Norwegian Data Inspectorate and the Regional Committee for Ethics in Medical Research (2010/2627-3).

## Results

Data from 20 338 women and 17 274 men aged 30–69 years were analysed in accordance with their self-reported, global perception of their childhood. In total, 85.4% of the respondents characterised their childhood as very good or good, 3.3% as difficult and 0.8% as very difficult (Table 1).

**Table 1:** Baseline characteristics of participants aged 30–69 years according to childhood experience in the HUNT Study (2006–8).

	Childhood experience:					
	Very good	Good	Average	Difficult	Very difficult	p trend*
**Number of participants**	17 759 (47.2)	14 351 (38.2)	3 993 (10.6)	1 225 (3.3)	284 (0.8)	Na
**Mean age**	50.9 (±10.6)	52.1 (±10.6)	51.3 (±10.5)	49.5 (±10.3)	47.6 (±10.3)	0.72
**Gender**						
**Female**	9 574 (53.9)	7 463 (52.0)	2 328 (58.3)	784 (64.0)	189 (66.5)	<0.001
**Male**	8 185 (46.1)	6 888 (48.0)	1 665 (41.7)	441 (36.0)	95 (33.5)	
**Daily smoking**	4 644 (26.2)	3 881 (26.6)	1 116 (27.9)	438 (35.8)	123 (43.7)	<0.001
**Mean nr of cigarettes**	11.7 (± 7.2)	12.1 (± 6.9)	12.7 (±7.5)	13.6 (±7.1)	15.7 (±10.3)	<0.001
**Insomnia**	3 159 (17.8)	3 168 (22.1)	1 131 (28.3)	442 (36.1)	113 (39.8)	<0.001
**Physical activity**						
**None**	332 (2.4)	263 (2.3)	91 (2.9)	50 (5.2)	17 (7.6)	<0.001
**Low**	3 191 (22.7)	2 765 (24.0)	789 (24.9)	237 (24.8)	66 (29.6)	
**Medium**	4 580 (32.6)	3 943 (34.3)	1 055 (33.3)	308 (32.3)	65 (29.2)	
**High**	5 949 (42.3)	4 528 (39.4)	1 229 (38.8)	360 (37.7)	75 (33.6)	
**Education**						
**Primary**	2 933 (21.3)	2 834 (25.5)	753 (25.7)	219 (27.4)	49 (34.8)	<0.001
**Secondary**	7 077 (51.4)	5 645 (50.8)	1 479 (50.6)	421 (52.6)	72 (51.1)	
**University**	3 754 (27.3)	2 632 (23.7)	693 (23.7)	160 (20.0)	20 (14.2)	

Standard deviation (SD) and percentages within brackets as appropriate.

*p trend calculated with ANOVA or Mantel-Haenszel test for linear association as appropriate.

In general, individuals reporting a difficult or a very difficult childhood were younger (p-trend significant when stratified by gender) and more often female. Smoking was more prevalent in this group and they reported higher cigarette consumption than smokers in other groups. They also reported more sleep problems, less physical activity and a lower educational level. A significant trend was observed from very good to very difficult childhood in all baseline characteristics except for age.

### Multimorbidity and childhood experience

**Fig 2:**
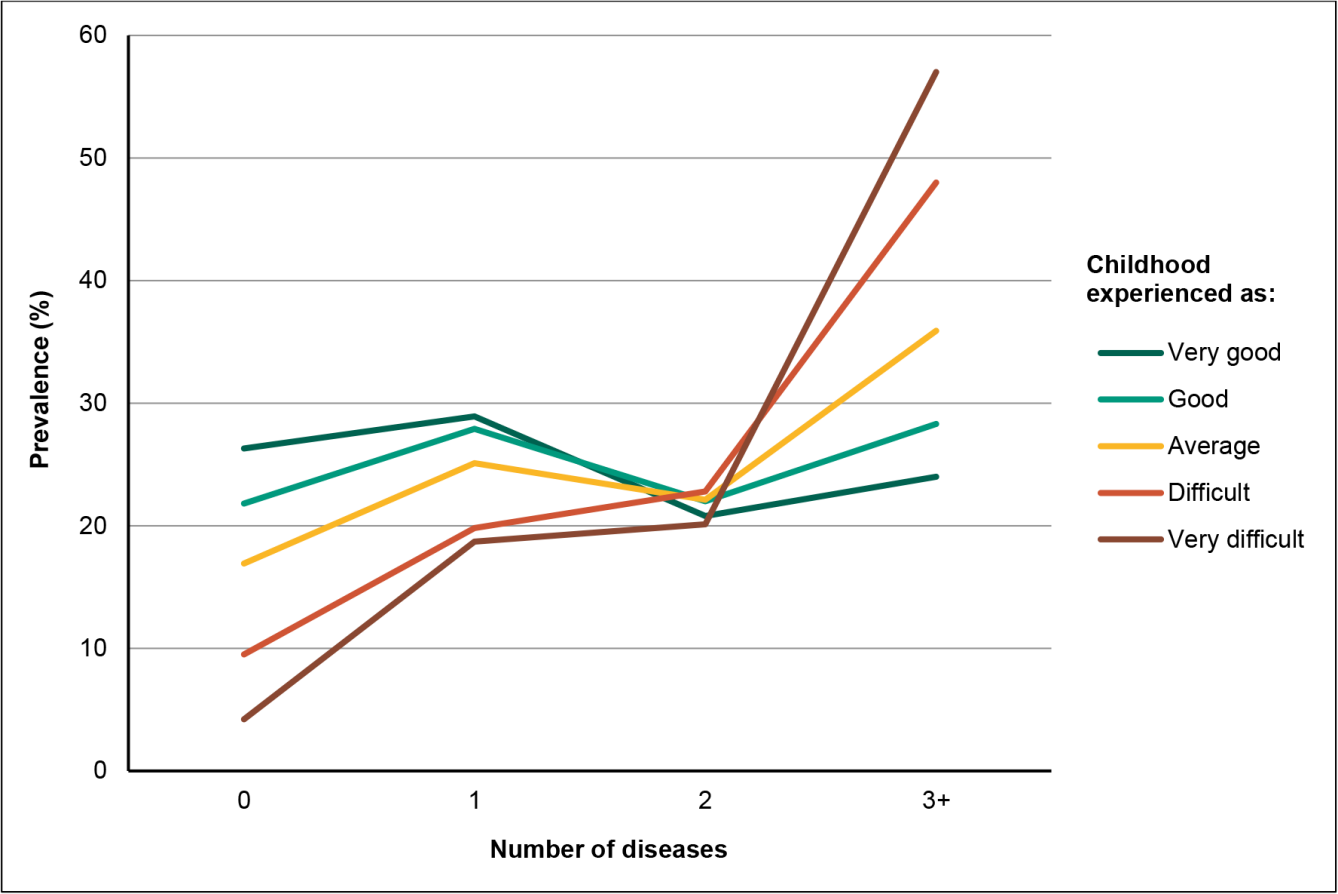
Number of diseases in adulthood (30–69y) according to childhood experience in the HUNT3 Study.

Fig 2 (and S1 Table) shows the *prevalence of number of diseases* for each given group. Respondents characterising their childhood as very good had a lower number of diseases, with 26.3% reporting no disease, compared to 9.5% and 4.2% for those reporting a difficult and a very difficult childhood, respectively. The total prevalence of multimorbidity increased from 44.8% among respondents reporting a very good childhood to 77.1% among those with a very difficult childhood. For individuals reporting a very difficult childhood, the age adjusted prevalence ratios gradually rose to 1.90, compared to those reporting a very good childhood.

**Fig 3:**
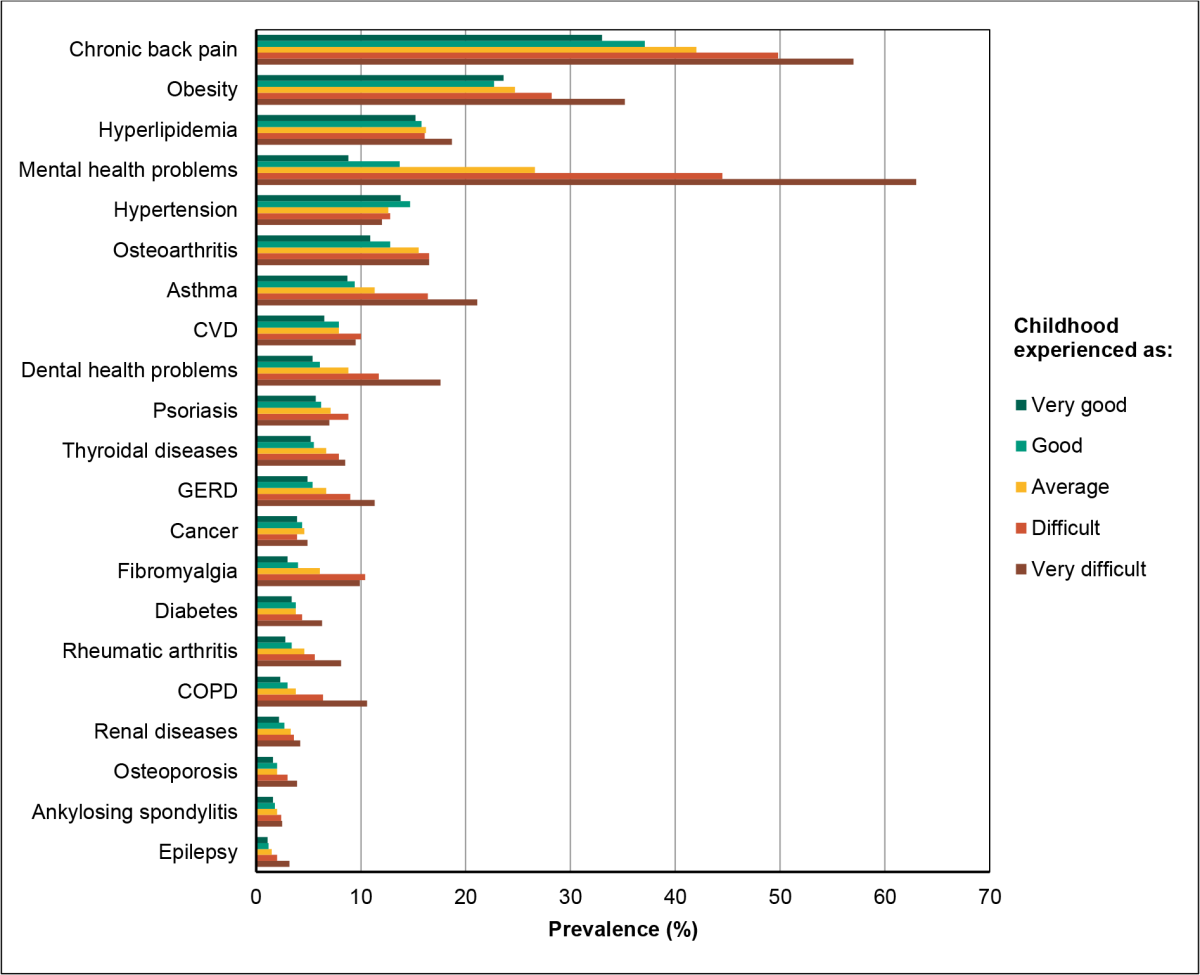
Prevalence of diseases/conditions according to childhood experience for adults (30–69y) in the HUNT3 Study.

A similar trend was found for the prevalence of *individual disease*s (Fig 3). The prevalence increased significantly with increasing degrees of childhood difficulty for all diseases, except hypertension and cancer. The increase was sevenfold for mental health problems, fourfold for chronic obstructive pulmonary disease (COPD) and dental health problems, and more than double for fibromyalgia, gastro-oesophageal reflux disease (GERD), rheumatic arthritis and asthma. The prevalence increased almost parallel in both genders, although the absolute prevalence of some diseases differed.

### Logistic regression analyses

In the first crude model which did not include any intervening factors, the OR of multimorbidity increased from 1.20 for those with a good childhood to 5.08 (95% CI 3.63–7.11) for individuals reporting a very difficult childhood, compared to very good childhood as reference (Table 2).

**Table 2:** Logistic models for multimorbidity according to childhood experience for participants aged 30–69 years in the HUNT Study (2006–8).

Childhood experience:										
	Very good		Good		Average		Difficult		Very difficult	
Logistic models	OR	95% CI	OR	95% CI	OR	95% CI	OR	95% CI	OR	95% CI
**Model 1**	1.0	Ref.	1.20	1.13–1.26	1.77	1.63–1.93	3.52	3.00–4.13	5.08	3.63–7.11
**Model 2**	1.0	Ref.	1.15	1.09–1.22	1.64	1.50–1.79	3.00	2.55–3.53	3.98	2.82–5.62
**Model 3**	1.0	Ref.	1.23	1.16–1.30	1.82	1.67–2.00	3.55	3.00–4.21	4.71	3.29–6.75
**Model 4**	1.0	Ref.	1.19	1.12–1.26	1.70	1.55–1.87	3.03	2.54–3.61	3.77	2.61–5.45

Odds ratios (OR) and 95% confidence intervals (95% CI) with very good childhood as a reference (Ref.).

Model 1: Adjusted for age and gender; Model 2: Adjusted for age, gender, smoking, insomnia, physical activity and education; Model 3: Adjusted for age, gender and allostatic factors; Model 4: Adjusted for all factors mentioned before.

The behavioural factors were then introduced one by one to evaluate their association with multimorbidity (S2 Table). Smoking, physical activity and educational level all lowered the OR marginally. The strongest single factor impact was found for sleep problems with OR declining from 5.08 to 4.32 (95% CI 3.07–6.07) for participants with a very difficult childhood.

Analysed individually, the allostatic parameters showed marginal or no impact on OR (S2 Table). When introduced to the model in combination (Table 2- Model 3) the OR associated with a very difficult childhood declined from 5.08 to 4.73 (95% CI 3.30–7.68) with no effect on OR for the other groups of childhood experience. Combined, the behavioural factors had a stronger impact on OR in very difficult childhood (OR 3.98, Model 3). When all behavioural and allostatic factors were combined, the OR declined to 3.78 (95% CI 2.61–5.47) (Model 4).

Adjusting for current depression in the crude model reduced the OR for very difficult childhood from 5.08 to 4.52 (95% CI 3.20–6.36). In the group with current depression, 11.1% reported a difficult or a very difficult childhood, compared to 4.1% in the group in general. The prevalences of different childhood qualities and multimorbidty did not differ significantly after excluding participants reporting current depression.

### Childhood experience and allostatic load

The mean values of eight of the 12 analysed allostatic parameters (Tables 3 and 4) differed according to the participants’ description of their childhood (p<0.05). Those reporting a difficult or very difficult childhood had, on average, shorter stature, larger waist circumference, higher waist hip ratio and BMI, higher resting heart rate, lower systolic blood pressure, and lower pulse pressure, compared to the other groups. Females but not males reporting a difficult childhood had significantly higher non-fasting blood glucose. Correspondingly, males but not females had a statistically significant trend towards lower diastolic blood pressure (Tables 3 and 4).

**Table 3:** Age adjusted difference from reference values of secondary allostatic parameters with 95% confidence intervals (95% CI) according to childhood experience among women aged 30–69 years, in the HUNT Study (2006–8) (N = 20 338).

	Childhood experience:					
	Very good	Good	Average	Difficult	Very difficult	
Women	Reference	Difference (95% CI)	Difference (95% CI)	Difference (95% CI)	Difference (95% CI)	p trend
Height (cm)	165.54	-0.02 (-0.20 to 0.15)	-0.65 (-0.91 to -0.38)	-0.69 (-1.12 to -0.27)	-1.71 (-2.54 to -0.87)	<0.001
Waist (cm)	90.36	-0.25 (-0.63 to 0.13)	0.20 (-0.37 to 0.76)	1.80 (0.89 to 2.70)	3.93 (2.15 to 5.72)	<0.001
WHR	0.87	0.00 (0.00 to 0.00)	0.00 (0.00 to 0.00)	0.01 (0.01 to 0.02)	0.02 (0.01 to 0.03)	<0.001
BMI (kg/m^2^)	27.01	-0.16 (-0.30 to -0.01)	0.21 (-0.01 to 0.42)	0.72 (0.37 to 1.07)	1.54 (0.85 to 2.23)	<0.001
SBP (mmHg)	124.87	-0.76 (-1.31 to -0.21)	-1.01 (-1.81 to -0.20)	-1.65 (-2.98 to -0.33)	0.63 (-1.99 to 3.26)	0.002
DBP (mmHg)	71.05	-0.50 (-0.85 to -0.15)	-0.11 (-0.63 to 0.41)	-0.49 (-1.34 to 0.36)	0.83 (-0.85 to 2.51)	0.26
Heart rate	71.04	0.13 (-0.25 to 0.50)	0.27 (-0.28 to 0.82)	0.44 (-0.46 to 1.35)	2.36 (0.59 to 4.14)	0.03
PP (mmHg)	91.82	-0.63 (-1.05 to -0.21)	-0.43 (-1.05 to 0.19)	-1.19 (-2.22 to -0.17)	1.08 (-0.94 to 3.09)	0.03
CRP (mg/L)	2.65	-0.01 (-0.20 to 0.19)	0.01 (-0.28 to 0.31)	0.47 (-0.01 to 0.95)	0.89 (-0.04 to 1.83)	0.08
Chol (mmol/L)	5.58	-0.01 (-0.04 to 0.02)	0.03 (-0.02 to 0.07)	0.03 (-0.04 to 0.11)	0.19 (0.04 to 0.34)	0.07
Glu (mmol/L)	5.31	-0.01 (-0.05 to 0.02)	0.00 (-0.05 to 0.05)	0.11 (0.03 to 0.18)	0.19 (0.04 to 0.34)	0.04
Crea (μmol/L)	75.81	0.08 (-0.36 to 0.51)	0.00 (-0.65 to 0.65)	0.38 (-0.68 to 1.43)	-0.31 (-2.39 to 1.76)	0.73

WHR = Waist hip ratio; BMI = Body mass index; SBP = Systolic blood pressure; DBP: Diastolic blood pressure; PP = Pulse pressure; CRP = C-reactive protein; Chol = S-Cholesterol; Glu = Non-fasting S-glucose; Crea = S-Creatinine.

**Table 4:** Age adjusted difference from reference values of secondary allostatic parameters with 95% confidence intervals (95% CI) according to childhood experience among men aged 30–69 years, in the HUNT Study (2006–8) (N = 17 274).

Childhood experience:						
	Very good	Good	Average	Difficult	Very difficult	
Men	Reference	Difference (95% CI)	Difference (95% CI)	Difference (95% CI)	Difference (95% CI)	p trend
Height (cm)	178.56	0.01 (-0.20 to 0.21)	-0.27 (-0.60 to 0.07)	-0.50 (-1.10 to 0.11)	-1.87 (-3.15 to -0.59)	0.001
Waist (cm)	97.58	0.09 (-0.24 to 0.41)	0.66 (0.12 to 1.19)	2.66 (1.69 to 3.63)	2.06 (0.01 to 4.11)	<0.001
WHR	0.94	0.00 (0.00 to 0.00)	0.01 (0.00 to 0.01)	0.02 (0.01 to 0.02)	0.02 (0.01 to 0.04)	<0.001
BMI (kg/m^2^)	27.72	-0.04 (-0.16 to 0.08)	0.07 (-0.13 to 0.27)	0.70 (0.34 to 1.06)	0.55 (-0.21 to 1.30)	0.01
SBP (mmHg)	131.76	-0.30 (-0.87 to 0.26)	-0.81 (-1.74 to 0.13)	-1.30 (-3.01 to 0.40)	-3.82 (-7.40 to -0.23)	0.007
DBP (mmHg)	77.19	-0.23 (-0.61 to 0.14)	-0.65 (-1.27 to -0.03)	-0.24 (-1.37 to 0.89)	-2.52 (-4.89 to -0.14)	0.01
Heart rate	67.80	0.35 (-0.06 to 0.77)	0.07 (-0.62 to 0.75)	3.07 (1.82 to 4.32)	0.84 (-1.80 to 3.47)	<0.001
PP (mmHg)	97.16	-0.17 (-0.62 to 0.29)	-0.70 (-1.45 to 0.06)	-0.21 (-1.58 to 1.16)	-3.10 (-5.98 to -0.22)	0.04
CRP (mg/L)	2.37	0.00 (-0.19 to 0.19)	0.05 (-0.26 to 0.37)	0.64 (0.06 to 1.21)	0.15 (-1.05 to 1.36)	0.19
Chol (mmol/L)	5.53	0.01 (-0.02 to 0.05)	0.00 (-0.05 to 0.06)	0.02 (-0.08 to 0.12)	0.22 (0.01 to 0.43)	0.24
Glu (mmol/L)	5.56	-0.01 (-0.05 to 0.04)	0.00 (-0.07 to 0.06)	0.20 (0.08 to 0.33)	0.12 (-0.15 to 0.39)	0.11
Crea (μmol/L)	90.10	-0.07 (-0.61 to 047)	0.48 (-1.37 to 0.41)	0.53 (-1.10 to 2.17)	-1.80 (-5.23 to 1.64)	0.48

WHR = Waist hip ratio; BMI = Body mass index; SBP = Systolic blood pressure; DBP: Diastolic blood pressure; PP = Pulse pressure; CRP = C-reactive protein; Chol = S-Cholesterol; Glu = Non-fasting S-glucose; Crea = S-Creatinine.

## Discussion

Based on data from a large, stable and relatively affluent Norwegian population, we have documented a clear association between self-reported childhood difficulties and adult disease burden. With increasing childhood difficulties, the prevalence of multimorbidity, as well as most of the eligible diseases and disorders, increased in a dose-response manner. Sleep problems, physical activity and smoking habits followed a similar trend. The cross-sectional study design does not permit direct, causal inferences. Our findings are however concordant with an increasing body of evidence which links childhood adversities to ill health in a life-course perspective [70–72].

The fact that one question about subjective childhood experience gave such could yield such results, is a new finding. The approach needs further validation in other contexts, but might ultimately prove to have certain qualities in common with the single item questions about self-rated health [73].

Since this is a cross-sectional study, recall bias connected to the respondents’ childhood cannot be ruled out. A heavy disease burden might theoretically be blamed on childhood adversities. Previous studies which have compared retrospective and prospective data on childhood adversity have however not found evidence of recall bias [49,74,75]. The possibility is further diminished as we adjusted for current depression.

Approximately four percent of the HUNT3 study participants reported a difficult or very difficult childhood. This number is low, if compared to those that have focused on specified types of adverse events in childhood [52,53,76,77]. Our global experience question is obviously different, as it addresses the respondent’s personal appraisal of what might be described as the overall balance between adverse (“drain”) and supporting and resilience (“gain”) factors [78] in childhood. The low figure might also reflect the relatively favourable socioeconomic conditions in North-Trøndelag population. A direct link between severe poverty in childhood, biological perturbations and disease in adult life has been found in several populations, including the Norwegian county Finnmark in the years 1890–1967 [79]. It is highly unlikely that reported childhood difficulties in HUNT3 refer to food shortage or poor housing on a comparable scale.

One important factor that can´t be evaluated in our study is the impact of parental health. Common genetic disease susceptibilities remain a potential bias that would most likely be of variable importance across the spectrum of diseases.

Concerning the measured allostatic load parameters, eight of the 12 showed an association with childhood experience. This is not surprising, as allostatic parameters are likely to change during the life-course, and we applied measurements performed in adulthood. Furthermore, not all parameters could be optimally evaluated (see methods section). Exclusion of respondents who reported a clinician-diagnosed (and thus presumably treated) diabetes and/or medicated hypertension should lead to underestimation of serum glucose and blood pressure levels. The same applies to cholesterol, as some respondents might have been taking cholesterol-lowering drugs.

The rise in individual disease prevalence with increasing childhood difficulties varied considerably in our study, but the general trend was a dose-response association. The slope was steepest for pain conditions and mental health problems, in accord with previous studies on the health impact of childhood adversity [45,48,80–82] and compatible with a recent study on the relationship between self-rated health and allostatic load in the HUNT population [83]. The trend was also present regarding a number of conditions where physiological dysregulation and life-style are known to interact and even enhance each other, such as obesity, diabetes, dental problems, asthma, COPD, and GERD [42,54,76,84,85]. We did not find any dose-response relationship for hypertension in our study. Some studies indicate an association between childhood adversities and hypertension [85], but this association may be complex, as blunting of the HPA-axis can occur over time, resulting in flattening of the diurnal cortisol rhythm [40,86–88].

As the HUNT Study was conceived in accordance with the traditional biomedical focus on single disease conditions according to the ‘silo’ model [19], both the researchers who designed the survey and the questionnaire respondents were ‘blinded’ to the research question of the present study. Consequently, expectation bias can be ruled out. The fact that diagnoses are self-reported, in contrast to studies based on medical records, can be considered both a weakness and strength, depending on the chosen perspective.

The fact that the HUNT population is ethnically homogenous, with high and socially equitable access to primary healthcare [65], might be considered a strength, as it documents that multimorbidity is a ubiquitous phenomenon in contemporary Western societies, not only related to social deprivation.

Socioeconomic status has a well documented link to multimorbidity, as previously mentioned [8, 16]. The lack of comprehensive SES data represents a clear weekness of our study. However, the County of North-Trøndelag has been a stable community with a less steep social gradient than many other populations [65].

A general weakness of the HUNT3 study is the limited participation rate, which must nevertheless be seen as acceptable in a contemporary international context, especially for the age groups included in the current analysis. Participation rates were lowest in the youngest and oldest age groups, especially for young males. It is, however, relevant to notice that younger participants generally reported a higher prevalence of a very difficult childhood than older participants. This might lead to underestimation of the total multimorbidity count in the population. Furthermore, a comparison between participants and non-participants in the HUNT3 study showed that non-participants tended to have a higher prevalence of index diseases as well as a higher mortality [64,66]. In total, our study probably underestimates the disease burden in the overall population.

## Conclusions and implications

Based on data from a general and relatively affluent Norwegian population, we have documented a general, graded association between self-reported childhood difficulties on the one hand and multimorbidity, individual disease burden and biological perturbations on the other. The finding is in accordance with an increasing body of research which conceptualises allostatic overload as an important route by which childhood adversities become biologically embodied [89]. Consequently, we argue that future research on the aetiology and demanding clinical management of multimorbidity [90] should direct more attention to the biological impact of the patients’ life experiences [23].

From the perspective of childhood adversity research, our study applied an original one-item “childhood experience question”. The finding of a strong relation between self-reported childhood difficulties and adult disease burden indicates that this approach can have considerable epidemiological and clinical relevance, worthy of further investigation.

## Supporting Information

S1 Table Gender specific prevalence of multimorbidity and age adjusted prevalence ratios (PR) with 95% confidence intervals (95% CI), associated with childhood experience in the HUNT Study (2006–8) (N = 37 612). (DOCX)

S2 Table Odds ratios (OR) with 95% confidence intervals (CI) of developing multimorbidity according to childhood experience for participants aged 30–69 years in the HUNT Study (2006–8) with very good childhood as a reference (Ref). All anlyses adjusted for age and gender and then according to different possible behavioural and allostatic factors.
(DOCX)

## References

[1] Salisbury C, Johnson L, Purdy S, Valderas JM, Montgomery AA. Epidemiology and impact of multimorbidity in primary care: a retrospective cohort study. Br J Gen Pract. 2011;61(582):e12–21. 10.3399/bjgp11X548929 21401985 PMC3020068

[2] Starfield B, Lemke KW, Bernhardt T, Foldes SS, Forrest CB, Weiner JP. Comorbidity: implications for the importance of primary care in 'case' management. Ann Fam Med. 2003;1(1):8–14. 15043174 10.1370/afm.1 PMC1466556

[3] World Health Organization The World Health Report 2008: Primary Health Care—Now more than ever New York: The World Health Organization; 2008 Available: http://www.who.int/whr/2008/en/.

[4] Uijen AA, van de Lisdonk EH. Multimorbidity in primary care: prevalence and trend over the last 20 years. Eur J Gen Pract. 2008;14 Suppl 1:28–32. 10.1080/13814780802436093 18949641

[5] Ward BW, Schiller JS. Prevalence of multiple chronic conditions among US adults: estimates from the National Health Interview Survey, 2010. Prev Chronic Dis. 2013;10:E65 10.5888/pcd10.120203 23618545 PMC3652717

[6] Institute of Medicine. Living Well with Chronic Illness—A call for Pulblic Health Action The US Institute of Medicine, 2012.

[7] van den Akker M, Buntinx F, Metsemakers JF, Roos S, Knottnerus JA. Multimorbidity in general practice: prevalence, incidence, and determinants of co-occurring chronic and recurrent diseases. J Clin Epidemiol. 1998;51(5):367–375. 9619963 10.1016/s0895-4356(97)00306-5

[8] Barnett K, Mercer SW, Norbury M, Watt G, Wyke S, Guthrie B. Epidemiology of multimorbidity and implications for health care, research, and medical education: a cross-sectional study. Lancet. 2012;380(9836):37–43. 10.1016/S0140-6736(12)60240-2 22579043

[9] Fortin M, Stewart M, Poitras ME, Almirall J, Maddocks H. A systematic review of prevalence studies on multimorbidity: toward a more uniform methodology. Ann Fam Med. 2012;10(2):142–151. 10.1370/afm.1337 22412006 PMC3315131

[10] Tomasdottir MO, Getz L, Sigurdsson JA, Petursson H, Kirkengen AL, Krokstad S, et al Co-and multimorbidity patterns in an unselected Norwegian population: cross-sectional analysis based on the HUNT Study and theoretical reflections concerning basic medical models. Eur J Pers Cent Healthc. 2014 2(3):335–345.

[11] Holden L, Scuffham PA, Hilton MF, Muspratt A, Ng SK, Whiteford HA. Patterns of multimorbidity in working Australians. Popul Health Metr. 2011;9(1):15 10.1186/1478-7954-9-15 21635787 PMC3123553

[12] Prados-Torres A, Poblador-Plou B, Calderon-Larranaga A, Gimeno-Feliu LA, Gonzalez-Rubio F, Poncel-Falco A, et al Multimorbidity patterns in primary care: interactions among chronic diseases using factor analysis. PloS One. 2012;7(2):e32190 10.1371/journal.pone.0032190 22393389 PMC3290548

[13] Schafer I, Hansen H, Schon G, Hofels S, Altiner A, Dahlhaus A, et al The influence of age, gender and socio-economic status on multimorbidity patterns in primary care. First results from the multicare cohort study. BMC Health Serv Res. 2012;12:89 10.1186/1472-6963-12-89 22471952 PMC3348059

[14] Fortin M, Bravo G, Hudon C, Vanasse A, Lapointe L. Prevalence of multimorbidity among adults seen in family practice. Ann Fam Med. 2005;3(3):223–228. 15928225 10.1370/afm.272 PMC1466875

[15] Tucker-Seeley RD, Li Y, Sorensen G, Subramanian SV. Lifecourse socioeconomic circumstances and multimorbidity among older adults. BMC Public Health. 2011;11:313 10.1186/1471-2458-11-313 21569558 PMC3118239

[16] Mercer SW, Guthrie B, Furler J, Watt GC, Hart JT. Multimorbidity and the inverse care law in primary care. BMJ. 2012;344:e4152 10.1136/bmj.e4152 22718915

[17] Smith SM, Soubhi H, Fortin M, Hudon C, O'Dowd T. Managing patients with multimorbidity: systematic review of interventions in primary care and community settings. BMJ. 2012;345:e5205 10.1136/bmj.e5205 22945950 PMC3432635

[18] Mercer SW, Smith SM, Wyke S, O'Dowd T, Watt GC. Multimorbidity in primary care: developing the research agenda. Fam Pract. 2009;26(2):79–80. 10.1093/fampra/cmp020 19287000

[19] Parekh AK, Barton MB. The challenge of multiple comorbidity for the US health care system. JAMA. 2010;303(13):1303–1304. 10.1001/jama.2010.381 20371790

[20] Mangin D, Heath I, Jamoulle M. Beyond diagnosis: rising to the multimorbidity challenge. BMJ. 2012;344:e3526 10.1136/bmj.e3526 22695898

[21] Miles A, Mezzich JE. The care of the patient and the soul of the clinic: person-centered medicine as an emergent model of modern medical practice. Int J Pers Cent Med. 2011;1:207–222.

[22] Starfield B. Is patient-centered care the same as person-focused care? Perm J. 2011;15(2):63–69. 21841928 10.7812/tpp/10-148 PMC3140752

[23] McEwen BS, Getz L. Lifetime experiences, the brain and personalized medicine: an integrative perspective. Metab. 2013;62 Suppl 1:S20–26. 10.1016/j.metabol.2012.08.020 23009787

[24] Barnes PJ. Chronic obstructive pulmonary disease: effects beyond the lungs. PLoS Med. 2010;7(3):e1000220 10.1371/journal.pmed.1000220 20305715 PMC2838746

[25] Kiecolt-Glaser JK, Gouin JP, Hantsoo L. Close relationships, inflammation, and health. Neurosci Biobehav Rev. 2010;35(1):33–38. 10.1016/j.neubiorev.2009.09.003 19751761 PMC2891342

[26] Stuart MJ, Baune BT. Depression and type 2 diabetes: inflammatory mechanisms of a psychoneuroendocrine co-morbidity. Neurosci Biobehav Rev. 2012;36(1):658–676. 10.1016/j.neubiorev.2011.10.001 22020230

[27] Halaris A. Inflammation, heart disease, and depression. Curr Psychiatry Rep. 2013;15(10):400 10.1007/s11920-013-0400-5 23975043

[28] Thayer JF, Yamamoto SS, Brosschot JF. The relationship of autonomic imbalance, heart rate variability and cardiovascular disease risk factors. Int J Cardiol. 2010;141(2):122–131. 10.1016/j.ijcard.2009.09.543 19910061

[29] Boer-Martins L, Figueiredo VN, Demacq C, Martins LC, Consolin-Colombo F, Figueiredo MJ, et al Relationship of autonomic imbalance and circadian disruption with obesity and type 2 diabetes in resistant hypertensive patients. Cardiovasc Diabetol. 2011;10:24 10.1186/1475-2840-10-24 21426540 PMC3072316

[30] Vinik AI, Maser RE, Ziegler D. Autonomic imbalance: prophet of doom or scope for hope? Diabet Med. 2011;28(6):643–651. 10.1111/j.1464-5491.2010.03184.x 21569084 PMC3123705

[31] Emin O, Esra G, Aysegul D, Ufuk E, Ayhan S, Rusen DM. Autonomic nervous system dysfunction and their relationship with disease severity in children with atopic asthma. Respir Physiol Neurobiol. 2012;183(3):206–210. 10.1016/j.resp.2012.07.002 22789502

[32] Pesonen AK, Raikkonen K, Feldt K, Heinonen K, Osmond C, Phillips DI, et al Childhood separation experience predicts HPA axis hormonal responses in late adulthood: a natural experiment of World War II. Psychoneuroendocrinology. 2010;35(5):758–767. 10.1016/j.psyneuen.2009.10.017 19963324

[33] Lovallo WR, Farag NH, Sorocco KH, Cohoon AJ, Vincent AS. Lifetime adversity leads to blunted stress axis reactivity: studies from the Oklahoma Family Health Patterns Project. Biol Psychiatry. 2012;71(4):344–349. 10.1016/j.biopsych.2011.10.018 22112928 PMC3264696

[34] Ehlert U. Enduring psychobiological effects of childhood adversity. Psychoneuroendocrinology. 2013;38(9):1850–1857. 10.1016/j.psyneuen.2013.06.007 23850228

[35] McGowan PO, Szyf M. The epigenetics of social adversity in early life: implications for mental health outcomes. Neurobiol Dis. 2010;39(1):66–72. 10.1016/j.nbd.2009.12.026 20053376

[36] Feinberg AP. The epigenetic basis of common human disease. Trans Am Clin Climatol Assoc. 2013;124:84–93. 23874013 PMC3715917

[37] Blackburn EH, Epel ES. Telomeres and adversity: Too toxic to ignore. Nature. 2012;490(7419):169–171. 10.1038/490169a 23060172

[38] Needham BL, Adler N, Gregorich S, Rehkopf D, Lin J, Blackburn EH, et al Socioeconomic status, health behavior, and leukocyte telomere length in the National Health and Nutrition Examination Survey, 1999–2002. Soc Sci Med. 2013;85:1–8. 10.1016/j.socscimed.2013.02.023 23540359 PMC3666871

[39] McEwen BS. Protective and damaging effects of stress mediators. N Engl J Med. 1998;338(3):171–179. 9428819 10.1056/NEJM199801153380307

[40] McEwen BS, Wingfield JC. The concept of allostasis in biology and biomedicine. Horm Behav. 2003;43(1):2–15. 12614627 10.1016/s0018-506x(02)00024-7

[41] McEwen BS. Protective and damaging effects of stress mediators: central role of the brain. Dialogues Clin Neurosci. 2006;8(4):367–381. 17290796 10.31887/DCNS.2006.8.4/bmcewen PMC3181832

[42] ACE. The Adverse Childhood Experiences Study homepage. Available: www.cdc.gov/ace/.

[43] Felitti VJ, Anda RF, Nordenberg D, Williamson DF, Spitz AM, Edwards V, et al Relationship of childhood abuse and household dysfunction to many of the leading causes of death in adults. The Adverse Childhood Experiences (ACE) Study. Am J Prev Med. 1998;14(4):245–258. 9635069 10.1016/s0749-3797(98)00017-8

[44] McEwen BS. Sleep deprivation as a neurobiologic and physiologic stressor: Allostasis and allostatic load. Metab. 2006;55(10 Suppl 2):S20–23. 10.1016/j.metabol.2006.07.008 16979422

[45] Sachs-Ericsson N, Cromer K, Hernandez A, Kendall-Tackett K. A review of childhood abuse, health, and pain-related problems: the role of psychiatric disorders and current life stress. J Trauma Dissociation. 2009;10(2):170–188. 10.1080/15299730802624585 19333847

[46] Kirkengen AL. The Lived Experience of Violation: How Abused Children Become Unhealthy Adults Bucharest: Zeta Books; 2010.

[47] Cuijpers P, Smit F, Unger F, Stikkelbroek Y, Ten Have M, de Graaf R. The disease burden of childhood adversities in adults: a population-based study. Child Abuse Negl. 2011;35(11):937–945. 10.1016/j.chiabu.2011.06.005 22099144

[48] Gonzalez A, Boyle MH, Kyu HH, Georgiades K, Duncan L, MacMillan HL. Childhood and family influences on depression, chronic physical conditions, and their comorbidity: findings from the Ontario Child Health Study. J Psychiatr Res. 2012;46(11):1475–1482. 10.1016/j.jpsychires.2012.08.004 22959202

[49] Rich-Edwards JW, Mason S, Rexrode K, Spiegelman D, Hibert E, Kawachi I, et al Physical and sexual abuse in childhood as predictors of early-onset cardiovascular events in women. Circulation. 2012;126(8):920–927. 10.1161/CIRCULATIONAHA.111.076877 22787111 PMC3649533

[50] Brent DA, Silverstein M. Shedding light on the long shadow of childhood adversity. JAMA. 2013;309(17):1777–1778. 10.1001/jama.2013.4220 23632718 PMC3997256

[51] Das-Munshi J, Clark C, Dewey ME, Leavey G, Stansfeld SA, Prince MJ. Does childhood adversity account for poorer mental and physical health in second-generation Irish people living in Britain? Birth cohort study from Britain (NCDS). BMJ open. 2013;3(3). 10.1136/bmjopen-2012-001335 PMC3612813 23457320

[52] Kelly-Irving M, Lepage B, Dedieu D, Bartley M, Blane D, Grosclaude P, et al Adverse childhood experiences and premature all-cause mortality. Eur J Epidemiol. 2013;28(9):721–734. 10.1007/s10654-013-9832-9 23887883 PMC3787798

[53] Mason SM, Flint AJ, Field AE, Austin SB, Rich-Edwards JW. Abuse victimization in childhood or adolescence and risk of food addiction in adult women. Obesity. 2013;21(12):E775–781. 10.1002/oby.20500 23637085 PMC3855159

[54] Lee C, Tsenkova V, Carr D. Childhood trauma and metabolic syndrome in men and women. Soc Sci Med. 2014;105c:122–130. 10.1016/j.socscimed.2014.01.017 PMC4097386 24524907

[55] Garner AS, Shonkoff JP. Early childhood adversity, toxic stress, and the role of the pediatrician: translating developmental science into lifelong health. Pediatrics. 2012;129(1):e224–231. 10.1542/peds.2011-2662 22201148

[56] Carlsson E, Frostell A, Ludvigsson J, Faresjo M. Psychological stress in children may alter the immune response. J Immunol. 2014;192(5):2071–2081. 10.4049/jimmunol.1301713 24501202

[57] Savolainen K, Eriksson JG, Kananen L, Kajantie E, Pesonen AK, Heinonen K, et al Associations between early life stress, self-reported traumatic experiences across the lifespan and leukocyte telomere length in elderly adults. Biol Psychol. 2014;97:35–42. 10.1016/j.biopsycho.2014.02.002 24530884

[58] Starkweather AR, Alhaeeri AA, Montpetit A, Brumelle J, Filler K, Montpetit M, et al An integrative review of factors associated with telomere length and implications for biobehavioral research. Nurs Res. 2014;63(1):36–50. 10.1097/NNR.0000000000000009 24335912 PMC4112289

[59] Blair C, Raver CC, Granger D, Mills-Koonce R, Hibel L. Allostasis and allostatic load in the context of poverty in early childhood. Dev Psychopathol. 2011;23(3):845–857. 10.1017/S0954579411000344 21756436 PMC4167021

[60] Shonkoff JP, Boyce WT, McEwen BS. Neuroscience, molecular biology, and the childhood roots of health disparities: building a new framework for health promotion and disease prevention. JAMA. 2009;301(21):2252–2259. 10.1001/jama.2009.754 19491187

[61] Shonkoff JP, Garner AS. The lifelong effects of early childhood adversity and toxic stress. Pediatrics. 2012;129(1):e232–246. 10.1542/peds.2011-2663 22201156

[62] Danese A, McEwen BS. Adverse childhood experiences, allostasis, allostatic load, and age-related disease. Physiol Beha. 2012;106(1):29–39. 10.1016/j.physbeh.2011.08.019 21888923

[63] Gruenewald TL, Karlamangla AS, Hu P, Stein-Merkin S, Crandall C, Koretz B, et al History of socioeconomic disadvantage and allostatic load in later life. Soc Sci Med. 2012;74(1):75–83. 10.1016/j.socscimed.2011.09.037 22115943 PMC3264490

[64] Krokstad S, Langhammer A, Hveem K, Holmen T, Midthjell K, Stene T, et al Cohort Profile: The HUNT Study, Norway. Int J Epidemiol. 2013;42(4):968–977. 10.1093/ije/dys095 22879362

[65] Vikum E, Krokstad S, Westin S. Socioeconomic inequalities in health care utilisation in Norway: the population-based HUNT3 survey. Int J Equity Health. 2012;11:48 10.1186/1475-9276-11-48 22909009 PMC3490903

[66] Langhammer A, Krokstad S, Romundstad P, Heggland J, Holmen J. The HUNT study: participation is associated with survival and depends on socioeconomic status, diseases and symptoms. BMC Med Res Methodol. 2012;12:143 10.1186/1471-2288-12-143 22978749 PMC3512497

[67] Beckie TM. A systematic review of allostatic load, health, and health disparities. Biol Res Nurs. 2012;14(4):311–346. 23007870 10.1177/1099800412455688

[68] McEwen BS. Biomarkers for assessing population and individual health and disease related to stress and adaptation. Metab. 2015;64(3 Suppl 1):S2–s10. 10.1016/j.metabol.2014.10.029 25496803

[69] Juster RP, McEwen BS, Lupien SJ. Allostatic load biomarkers of chronic stress and impact on health and cognition. Neurosci Biobehav Rev. 2010;35(1):2–16. 10.1016/j.neubiorev.2009.10.002 19822172

[70] Barboza Solis C, Kelly-Irving M, Fantin R, Darnaudery M, Torrisani J, Lang T, et al Adverse childhood experiences and physiological wear-and-tear in midlife: Findings from the 1958 British birth cohort. Proc Natl Acad Sci U S A. 2015;112(7):E738–746. 10.1073/pnas.1417325112 25646470 PMC4343178

[71] Chapman DP, Wheaton AG, Anda RF, Croft JB, Edwards VJ, Liu Y, et al Adverse childhood experiences and sleep disturbances in adults. Sleep med. 2011;12(8):773–779. 10.1016/j.sleep.2011.03.013 21704556

[72] Widom CS, Horan J, Brzustowicz L. Childhood maltreatment predicts allostatic load in adulthood. Child Abuse Negl. 2015. 10.1016/j.chiabu.2015.01.016 PMC4539293 25700779

[73] Schnittker J, Bacak V. The increasing predictive validity of self-rated health. PloS One. 2014;9(1):e84933 10.1371/journal.pone.0084933 24465452 PMC3899056

[74] Rich-Edwards JW, Spiegelman D, Lividoti Hibert EN, Jun HJ, Todd TJ, Kawachi I, et al Abuse in childhood and adolescence as a predictor of type 2 diabetes in adult women. Am J Prev Med. 2010;39(6):529–536. 10.1016/j.amepre.2010.09.007 21084073 PMC3003936

[75] Hardt J, Vellaisamy P, Schoon I. Sequelae of prospective versus retrospective reports of adverse childhood experiences. Psychol Rep. 2010;107(2):425–440. 21117468 10.2466/02.04.09.10.16.21.PR0.107.5.425-440

[76] Korkeila J, Lietzen R, Sillanmaki LH, Rautava P, Korkeila K, Kivimaki M, et al Childhood adversities and adult-onset asthma: a cohort study. BMJ Open. 2012;2(5). 10.1136/bmjopen-2012-001625 PMC3488721 23069774

[77] Sethi D, Bellis M, Hughes K, Gilbert R, Mitis F, Galea G. European report on preventing child maltreatment Europe: World Health Organization, 2013.

[78] Karatsoreos IN, McEwen BS. Annual Research Review: The neurobiology and physiology of resilience and adaptation across the life course. J Child Psychol Psychiatry. 2013;54(4):337–347. 10.1111/jcpp.12054 23517425

[79] Forsdahl A. Observations throwing light on the high mortality in the county of Finnmark. Is the high mortality today a late effect of very poor living conditions in childhood and adolescence? Int J Epidemiol. 2002;31(2):302–308. 11980784

[80] Miller GE, Cole SW. Clustering of depression and inflammation in adolescents previously exposed to childhood adversity. Biol Psychiatry. 2012;72(1):34–40. 10.1016/j.biopsych.2012.02.034 22494534 PMC3493164

[81] Chaloner A, Greenwood-Van Meerveld B. Early life adversity as a risk factor for visceral pain in later life: importance of sex differences. Front Neurosci. 2013;7:13 10.3389/fnins.2013.00013 23407595 PMC3570767

[82] Fryers T, Brugha T. Childhood determinants of adult psychiatric disorder. Clin Pract Epidemiol Ment Health. 2013;9:1–50. 10.2174/1745017901309010001 23539489 PMC3606947

[83] Vie TL, Hufthammer KO, Holmen TL, Meland E, Breidablik HJ. Is self-rated health a stable and predictive factor for allostatic load in early adulthood? Findings from the Nord Trondelag Health Study (HUNT). Soc Sci Medicine. 2014;117c:1–9. 10.1016/j.socscimed.2014.07.019 25016460

[84] Boyce WT, Den Besten PK, Stamperdahl J, Zhan L, Jiang Y, Adler NE, et al Social inequalities in childhood dental caries: the convergent roles of stress, bacteria and disadvantage. Soc Sci Med. 2010;71(9):1644–1652. 10.1016/j.socscimed.2010.07.045 20870333 PMC2954891

[85] Riley EH, Wright RJ, Jun HJ, Hibert EN, Rich-Edwards JW. Hypertension in adult survivors of child abuse: observations from the Nurses' Health Study II. J Epidemiol Community Health. 2010;64(5):413–418. 10.1136/jech.2009.095109 20445210 PMC3744368

[86] Wirtz PH, von Kanel R, Emini L, Ruedisueli K, Groessbauer S, Maercker A, et al Evidence for altered hypothalamus-pituitary-adrenal axis functioning in systemic hypertension: blunted cortisol response to awakening and lower negative feedback sensitivity. Psychoneuroendocrinology. 2007;32(5):430–436. 17433557 10.1016/j.psyneuen.2007.02.006

[87] Harris A, Seckl J. Glucocorticoids, prenatal stress and the programming of disease. Horm Behav. 2011;59(3):279–289. 10.1016/j.yhbeh.2010.06.007 20591431

[88] Tyrka AR, Walters OC, Price LH, Anderson GM, Carpenter LL. Altered response to neuroendocrine challenge linked to indices of the metabolic syndrome in healthy adults. Horm Metab Res. 2012;44(7):543–549. 10.1055/s-0032-1306342 22549400 PMC3580172

[89] Krieger N. Embodiment: a conceptual glossary for epidemiology. J Epidemiol Community Health. 2005;59(5):350–355. 15831681 10.1136/jech.2004.024562 PMC1733093

[90] Sturmberg JP. Caring for people with chronic disease: is 'muddling through' the best way to handle the multiple complexities? J Eval Clin Pract. 2012;18(6):1220–1225. 10.1111/j.1365-2753.2012.01882.x 22846042

