# Supplementary material for: Self Reported Childhood Difficulties, Adult Multimorbidity and Allostatic Load. A Cross-Sectional Analysis of the Norwegian HUNT Study
Source: PLoS One. 2015 Jun 18;10(6):e0130591. doi: 10.1371/journal.pone.0130591 (PMC4472345; doi:10.1371/journal.pone.0130591)
Supplement: S1 Table — (DOCX) [file pone.0130591.s001.docx]

| **Childhood experience:** | | | | | | | | | | | |
| --- | --- | --- | --- | --- | --- | --- | --- | --- | --- | --- | --- |
|  | **Very good** | |  | | **Good** |  | **Average** |  | **Difficult** |  | **Very difficult** |
| **Women** | | | | | | | | | | | |
| 0-1 diseases | 4990 (52.1) | |  | | 3444 (46.2) |  | 940 (40.4) |  | 222 (28.3) |  | 43 (22.7) |
| Multimorbidity | 4584 (47.9) | |  | | 4019 (53.8) |  | 1388 (59.6) |  | 562 (71.7) |  | 146 (77.3) |
| PR (95% CI) | 1.00 (Reference) | |  | | 1.09 (1.06-1.13) |  | 1.25 (1.21-1.30) |  | 1.59 (1.51-1.66) |  | 1.82 (1.69-1.97) |
| **Men** | | | | | | | | | | | |
| 0-1 diseases | 4821 (58.9) |  | | 3688 (53.5) | |  | 738 (44.3) |  | 136 (30.9) |  | 22 (23.2) |
| Multimorbidity | 3364 (41.1) |  | | 3200 (46.5) | |  | 927 (55.7) |  | 305 (69.2) |  | 73 (76.8) |
| PR (95% CI) | 1.00 (Reference) |  | | 1.08 (1.04-1.12) | |  | 1.30 (1.24-1.37) |  | 1.70 (1.59-1.81) |  | 2.01 (1.82-2.22) |
| **Total** | | | | | | | | | | | |
| 0-1 diseases | 9811 (55.2) | |  | | 7132 (49.7) |  | 1678 (42.0) |  | 358 (29.2) |  | 65 (22.9) |
| Multimorbidity | 7948 (44.8) | |  | | 7219 (50.3) |  | 2315 (58.0) |  | 867 (70.8) |  | 219 (77.1) |
| PR (95% CI) | 1.00 (Reference) | |  | | 1.08 (1.06-1.11) |  | 1.28 (1.24-1.32) |  | 1.64 (1.58-1.71) |  | 1.90 (1.79-2.02) |
