# Supplementary material for: Self Reported Childhood Difficulties, Adult Multimorbidity and Allostatic Load. A Cross-Sectional Analysis of the Norwegian HUNT Study
Source: PLoS One. 2015 Jun 18;10(6):e0130591. doi: 10.1371/journal.pone.0130591 (PMC4472345; doi:10.1371/journal.pone.0130591)
Supplement: S2 Table — All anlyses adjusted for age and gender and then according to different possible behavioural and allostatic factors. (DOCX) [file pone.0130591.s002.docx]

| **Childhood experience:** | | | | | | | | | | | | | | | | | | | | | | | | | |
| --- | --- | --- | --- | --- | --- | --- | --- | --- | --- | --- | --- | --- | --- | --- | --- | --- | --- | --- | --- | --- | --- | --- | --- | --- | --- |
|  | **Very good** | |  | **Good** | |  | | | **Average** | | | | |  | | **Difficult** | | | |  | **Very difficult** | | | | |
|  | **OR** | **95%CI** |  | **OR** | **95%CI** |  | | | **OR** | | | **95%CI** | |  | | **OR** | | **95%CI** | |  | **OR** | | | **95%CI** | |
| Basic model | 1.0 | Ref. |  | 1.20 | 1.13-1.26 | | |  | | | 1.77 | | 1.63-1.93 | |  | | 3.52 | | 3.00-4.13 | | |  | 5.08 | | 3.63-7.11 |
| **Models adjusted for:** | | |  |  |  | | |  | | |  | |  | |  | |  | |  | | |  |  | |  |
| Smoking | 1.0 | Ref. |  | 1.20 | 1.13-1.26 | |  | | | 1.77 | | | 1.62-1.93 | |  | | 3.45 | | 2.94-4.04 | | |  | 4.86 | | 3.47-6.81 |
| Insomnia | 1.0 | Ref. |  | 1.16 | 1.10-1.23 | |  | | | 1.64 | | | 1.50-1.79 | |  | | 3.07 | | 2.61-3.61 | | |  | 4.32 | | 3.07-6.07 |
| Physical activity | 1.0 | Ref. |  | 1.19 | 1.13-1.26 | |  | | | 1.78 | | | 1.63-1.94 | |  | | 3.53 | | 3.01-4.15 | | |  | 5.03 | | 3.58-7.07 |
| Education | 1.0 | Ref. |  | 1.18 | 1.13-1.25 | |  | | | 1.74 | | | 1.60-1.90 | |  | | 3.41 | | 2.91-4.00 | | |  | 4.78 | | 3.42-6.69 |
| **Allostatic factors:** | | | | | | | | | | | | | | | | | | | | | | | | | |
| Height | 1.0 | Ref. |  | 1.20 | 1.13-1.26 | |  | | | 1.76 | | | 1.61-1.92 | |  | | 3.50 | | 2.99-4.11 | | |  | 4.90 | | 3.50-6.85 |
| Waist | 1.0 | Ref. |  | 1.22 | 1.15-1.30 | |  | | | 1.84 | | | 1.68-2.01 | |  | | 3.60 | | 3.04-4.26 | | |  | 5.14 | | 3.60-7.34 |
| BMI | 1.0 | Ref. |  | 1.20 | 1.13-1.26 | |  | | | 1.77 | | | 1.63-1.93 | |  | | 3.52 | | 3.01-4.13 | | |  | 5.09 | | 3.64-7.12 |
| SBP | 1.0 | Ref. |  | 1.20 | 1.14-1.27 | |  | | | 1.80 | | | 1.65-1.96 | |  | | 3.63 | | 3.10-4.26 | | |  | 5.13 | | 3.66-7.18 |
| DBP | 1.0 | Ref. |  | 1.20 | 1.14-1.27 | |  | | | 1.79 | | | 1.65-1.95 | |  | | 3.60 | | 3.07-4.22 | | |  | 5.15 | | 3.68-7.22 |
| Pulse | 1.0 | Ref. |  | 1.20 | 1.14-1.27 | |  | | | 1.78 | | | 1.63-1.94 | |  | | 3.49 | | 2.98-4.09 | | |  | 5.00 | | 3.57-7.00 |
| Cholesterol | 1.0 | Ref. |  | 1.19 | 1.13-1.26 | |  | | | 1.77 | | | 1.62-1.93 | |  | | 3.51 | | 3.00-4.12 | | |  | 5.08 | | 3.63-7.10 |
| Glucose | 1.0 | Ref. |  | 1.20 | 1.14-1.27 | |  | | | 1.79 | | | 1.64-1.95 | |  | | 3.50 | | 2.98-4.11 | | |  | 4.98 | | 3.55-6.98 |
| CRP | 1.0 | Ref. |  | 1.20 | 1.13-1.26 | |  | | | 1.78 | | | 1.63-1.94 | |  | | 3.49 | | 2.98-4.09 | | |  | 4.98 | | 3.56-6.97 |
| Creatinine | 1.0 | Ref. |  | 1.20 | 1.13-1.26 | |  | | | 1.78 | | | 1.63-1.94 | |  | | 3.51 | | 3.00-4.12 | | |  | 5.14 | | 3.68-7.19 |

BMI = Body mass index; SBP = Systolic blood pressure; DBP: Diastolic blood pressure; CRP = C-reactive protein.
